# Use of desflurane during anesthesia for resection of extra-adrenal pheochromocytoma: a case report

**DOI:** 10.1186/s40981-018-0212-z

**Published:** 2018-10-18

**Authors:** Yutaka Oda, Takahisa Adachi, Ryushi Komatsu, Motoko Shimada, Yukio Tanaka

**Affiliations:** 1 Department of Anesthesiology, Osaka City Juso Hospital, 2-12-27, Nonaka-kita, Yodogawa-ku, Osaka, 532-0034 Japan; 2 Department of Urology, Osaka City Juso Hospital, 2-12-27, Nonaka-kita, Yodogawa-ku, Osaka, 532-0034 Japan; 3 Department of Cardiology, Osaka City Juso Hospital, 2-12-27, Nonaka-kita, Yodogawa-ku, Osaka, 532-0034 Japan

**Keywords:** Desflurane, Extra-adrenal pheochromocytoma, ^123^Iodine-metaiodobenzylguanidine, Remifentanil

## Abstract

**Background:**

Although various agents are used during anesthesia for surgical resection of pheochromocytoma, application of desflurane has rarely been reported. We report the use of desflurane in a case receiving resection of extra-adrenal pheochromocytoma.

**Case presentation:**

A 51-year-old female was transferred to our hospital for sustained hypertension. A diagnosis of extra-adrenal pheochromocytoma was made based on increased plasma catecholamine levels and radiographic findings. Surgical resection was planned after controlling blood pressure. General anesthesia was induced with propofol and fentanyl, followed by maintenance with desflurane 4.3% and remifentanil 0.2–0.4 μg/kg/min. Blood pressure and heart rate were stable during induction, tracheal intubation, and tumor manipulation. Blood pressure abruptly decreased to 62/40 mmHg after removal of the tumor, which was treated with noradrenaline. The surgery was completed uneventfully and the postoperative course was also uneventful.

**Conclusion:**

Desflurane was safely used in combination with remifentanil during anesthesia for resection of extra-adrenal pheochromocytoma.

## Background

Pheochromocytomas are rare, catecholamine-producing, neuroendocrine tumors originating from the adrenal medulla or from chromaffin cells in the sympathetic ganglia. Patients with pheochromocytomas often present with the classic triad of diaphoresis, headache, and palpitations and have hypertension. Surgical resection under general anesthesia is the only curative treatment [1]. Both anesthesia and surgery are stimuli that elicit a catecholaminergic crisis, and preanesthetic preparation and intraoperative management of hemodynamic conditions are required. Although various anesthetics have been used for surgery of pheochromocytomas, there has been no preoperative and intraoperative management that can totally prevent hemodynamic fluctuations [2].

Desflurane is characterized by rapid elimination and early recovery from anesthesia [3]. Although desflurane elicits sympathetic activation, hypertension, and tachycardia when its inspired concentration exceeds 1.0 minimum alveolar concentration (MAC) [4], it provides faster and tighter hemodynamic control than other volatile agents at lower than 1.0 MAC [5, 6], suggesting that desflurane might be safely used during anesthesia for resection of pheochromocytomas. However, there have been only a few reports describing anesthesia with desflurane in patients undergoing pheochromocytoma surgery [7–9]. We report the use of desflurane in a patient undergoing resection of extra-adrenal pheochromocytoma by laparotomy.

## Case presentation

### Preoperative management

**Fig. 1:**
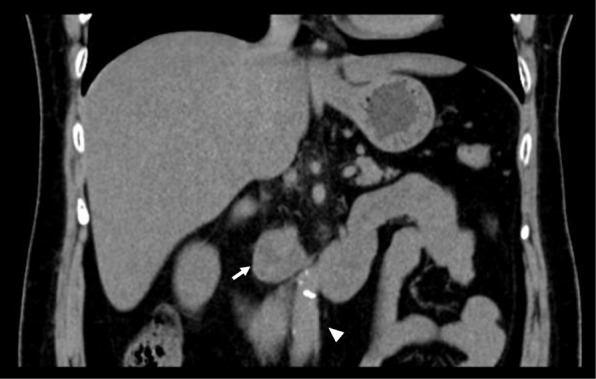
Coronal noncontrast computed tomography image. A heterogeneous mass in the right retroperitoneal region (white arrow) adjunct to the inferior vena cava (white arrow head) is demonstrated

We have obtained written informed consent from the patient for publication of this case report. A 51-year-old female weighing 61 kg was transferred to our hospital for sustained hypertension. She had been treated with a calcium channel blocker, an α- and β-adrenergic antagonist, and an angiotensin-converting enzyme inhibitor for 7 weeks. There were no symptoms of diaphoresis, headache, or palpitation. Her past history and family history were unremarkable. Physical examinations revealed no abnormal findings except hypertension (207/120 mmHg). Laboratory data were within normal limits, except increased serum noradrenaline (1.65 ng/mL, normal range 0.10–0.50 ng/mL) and adrenaline (0.11 ng/mL, normal range < 0.10 ng/mL). Serum dopamine, T3, T4, TSH, ACTH, cortisol, renin, and aldosterone levels were in the normal range. Echocardiography demonstrated mild left ventricular hypertrophy with reasonably well-preserved left ventricular function. Computed tomography revealed a heterogeneous mass measuring 31 × 22 mm in the right retroperitoneal space, adjacent to the inferior vena cava (Fig. 1). Scintigraphy identified an increased uptake of ^123^iodine-metaiodobenzylguanidine (^123^I-MIBG) corresponding to that mass, which lead to a diagnosis of extra-adrenal pheochromocytoma. Subsequently, administration of oral doxazosin 4 mg and bisoprolol 5 mg daily was commenced and the patient maintained blood pressure of approximately 150/70 mmHg. Doxazosin and bisoprolol were administered for 8 weeks, until the day of and 2 days before surgery, respectively.

### Anesthesia

**Fig. 2:**
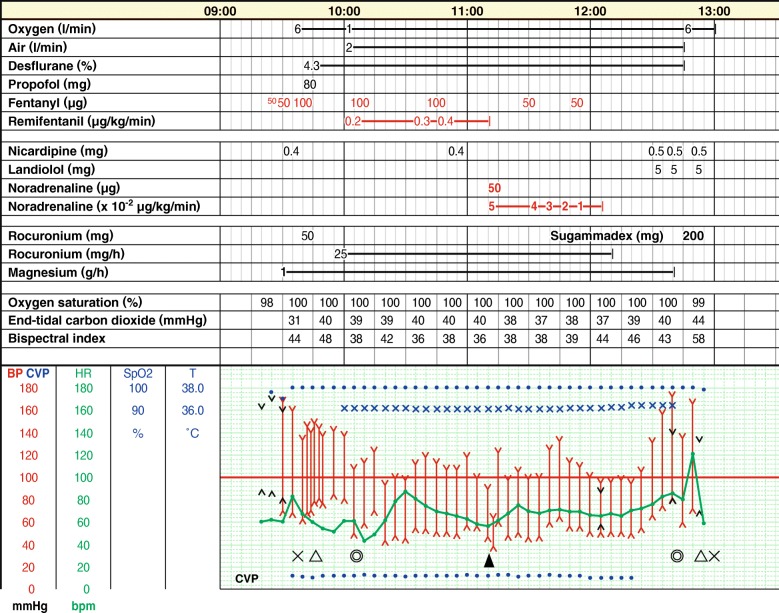
Anesthesia record. X, start and end of anesthesia; ∆, tracheal intubation and extubation; ◎, start and end of surgery; ▲, resection of the tumor

A central venous catheter was placed in the right jugular vein on the day before surgery. Crystalloid was infused at 100 mL/h during the 3 h of preoperative fasting period. Premedication was not used. Blood pressure was 160/90 mmHg and heart rate was 60/min on arrival at the operating room (Fig. 2). Fentanyl 100 μg was administered in two divided doses before cannulation of the left radial artery, followed by infusion of magnesium sulfate at 1 g/h. General anesthesia was induced with additional fentanyl 100 μg, propofol 80 mg, and rocuronium 50 mg while monitoring radial arterial pressure, central venous pressure (CVP), electrocardiogram, and peripheral oxygen saturation. Administration of desflurane 4.3% (age-adjusted 0.7 MAC) was started after tracheal intubation, and the lungs were ventilated with 45% oxygen and desflurane in order to maintain end-tidal carbon dioxide tension between 35 and 40 mmHg, which was continued until the end of surgery. Monitoring of cardiac output and stroke volume variation using FloTrac™ system (Edwards Life Sciences Corp., Irvine, CA, USA) was started after tracheal intubation. Continuous infusion of remifentanil 0.2 μg/kg/min was started, and fentanyl 100 μg bolus was administered respectively about 5 and 1 min before surgery. There was no hemodynamic response to tracheal intubation or surgical incision.

Blood pressure and heart rate remained stable throughout the initial period of surgery. The tumor was found to be adherent to the aorta and the inferior vena cava, necessitating manual dissection. Besides bolus administration of fentanyl 100 μg, infusion rate of remifentanil was increased to 0.4 μg/kg/min during direct tumor manipulation and was stopped following ligation of the draining vein. Blood pressure abruptly decreased to 62/40 mmHg immediately after tumor resection. A bolus infusion of noradrenaline 50 μg, followed by continuous infusion at 0.05 μg/kg/min, was started and blood pressure increased to 120/60 mmHg. Infusion rate of noradrenaline was decreased by 0.01 μg/kg/min and completely stopped before the end of surgery. CVP was maintained between 12 and 13 mmHg, and stroke volume variation was 9–11% throughout anesthesia.

Sugammadex 200 mg was administered on completion of surgery. Increases of blood pressure and heart rate were suppressed by three doses each of nicardipine 0.5 mg and landiolol 5 mg during emergence from anesthesia. Tracheal tube was removed after recovery of spontaneous respiration ≥ 12 breaths/min. The duration of surgery and anesthesia was 2 h 37 min and 3 h 25 min, respectively. Volume of infusion was 2500 mL, blood loss was 100 mL, and urine output was 200 mL. She was transferred to the high care unit, where postoperative analgesia was provided by intravenous patient-controlled analgesia with fentanyl 25 μg/h and acetaminophen 1000 mg every 8 h. She moved to the ward the next morning. Blood pressure was controlled around 160/90 mmHg with a calcium channel blocker and an angiotensin receptor blocker. She had an uneventful recovery with no abnormal laboratory data and was discharged home 10 days later.

## Discussion

The overall incidence of pheochromocytomas is 0.8 per 100,000 [10], and approximately 15–20% of them arise from the extra-adrenal chromaffin tissue [11]. Patients with retroperitoneal pheochromocytomas commonly present with symptoms similar to intra-adrenal pheochromocytomas such as diaphoresis, headache, and palpitations along with hypertension [12]. In the present case, hypertension refractory to medications was the only complaint. Increased plasma catecholamine levels, an extra-adrenal mass at computed tomography, and uptake of ^123^I-MIBG lead to the diagnosis. ^123^I-MIBG scintigraphy demonstrates high sensitivity and specificity to adrenergic tumors [13] and has been extensively applied in patients with clinical suspicion of pheochromocytoma. After antihypertensive medications for approximately 2 months, surgical resection was performed. Although laparoscopic removal is now the preferred surgical technique for both intra-adrenal and extra-adrenal pheochromocytomas [11], our case required laparotomy for predicted adhesion to the inferior vena cava.

The choice of anesthetic agent is generally less important than the depth of anesthesia for inhibiting adrenergic and cardiovascular responses [14]. Both inhalation and intravenous agents can be used, with an exception that morphine should be avoided due to its propensity to elicit histamine release [2, 15]. We induced general anesthesia with propofol and fentanyl, avoiding remifentanil because of possible hypotension during induction [16], followed by maintenance with 0.7 MAC desflurane throughout surgery, which is effective for controlling bispectral index below 50 [17]. Instead we adjusted the infusion rate of remifentanil according to the predictable degree of stimulation by tumor manipulation and resultant hemodynamic changes and were able to avoid hemodynamic responses to tracheal intubation and direct tumor manipulation. Although desflurane exerts sympathetic stimulation at an inspired concentration of 1.0–1.5 MAC [4], it is attenuated by a small dose of opioids [18]. Effective use of desflurane for controlling blood pressure during pheochromocytoma surgery and for rapid recovery from general anesthesia as a sole agent or in combination with remifentanil has been reported in a child as well as in adults [7–9].

We should note an increase of blood pressure and heart rate during emergence from anesthesia (Fig. 2). Although hypotension frequently occurs after removal of pheochromocytoma, hypertension is also a common complication [14]. According to a longtime follow-up study, approximately 50% of patients were hypertensive after resection of pheochromocytoma without recurrence [19], which was ascribed to underlying predisposing essential hypertension. Another study showed that peripheral sympathetic activity was significantly increased after pheochromocytoma surgery, despite decreased blood pressure, heart rate, and circulating catecholamine levels compared with those before surgery [20]. Hypertension is also reported immediately after surgery, which was attributed to postoperative pain [7]. Although the precise etiology of hypertension observed in our case is not clear, postoperative pain, preexisting essential hypertension, and increased peripheral sympathetic activity mediated by the brain during emergence from general anesthesia might contribute to it.

Evaluation of intravascular volume is essential during removal of pheochromocytomas. We used stroke volume variation for this purpose. It is calculated by continuous arterial pulse contour analysis during mechanical ventilation and is highly sensitive in estimating cardiac preload compared with CVP measurement [21]. Despite significant hypotension after removal of the tumor, stroke volume variation remained constant during surgery, suggesting that intravascular volume was largely unaffected.

Pheochromocytomas account for 0.3–0.6% of cases of hypertension [22, 23]. Despite improved diagnostic technique that can bring about an earlier diagnosis, there still usually remains a delay of 3 years between initial symptoms and the final diagnosis [24], probably resulting from non-specific symptoms. Prompt diagnosis and adequate treatment by the attending physicians and awareness of anesthesiologists regarding the possibility of pheochromocytomas, particularly in young patients with hypertension, would contribute to decreasing perioperative mortality and morbidity of patients undergoing surgery.

## Abbreviations

ACTH: Adrenocorticotropic hormone
CVP: Central venous pressure
MAC: Minimum alveolar concentration
MIBG: Metaiodobenzylguanidine
TSH: Thyroid-stimulating hormone
